# Substrate‐Controlled Response Coefficients in Thin Films

**DOI:** 10.1002/advs.202505761

**Published:** 2025-08-19

**Authors:** Marina Tyunina, Leonid L. Rusevich, Maxim Savinov, Tomas Kocourek, Oliva Pacherova, Alexandr Dejneka, Eugene A. Kotomin

**Affiliations:** ^1^ Microelectronics Research Unit Faculty of Information Technology and Electrical Engineering University of Oulu P. O. Box 4500 Oulu FI‐90014 Finland; ^2^ Institute of Physics of the Czech Academy of Sciences Na Slovance 2 Prague 18221 Czech Republic; ^3^ Institute of Solid State Physics University of Latvia Kengaraga Str. 8 Riga LV‐1063 Latvia; ^4^ Max Planck Institute for Solid State Research Heisenberg Str. 1 D‐70569 Stuttgart Germany

**Keywords:** computational methods, condensed matter physics, ferroelectrics, physics & engineering

## Abstract

To obtain materials with desired properties, material compositions are primarily altered, whereas thin films offer additional unique avenues. By combining state‐of‐the‐art first‐principles calculations and experimental investigations of thin films of strontium titanate as an exemplary representative of a broad class of perovskite oxides and the extensive family of ferroelectrics, a novel approach is presented to achieving superior material responses to external stimuli. The findings reveal that substrate‐imposed deformations, or strains, significantly alter the frequencies and magnitudes of atomic vibrations in thin films. Consequently, material‐specific response‐stimulus coefficients can become strain‐dependent. The strain‐dependent Curie constant, which characterizes the dielectric response to thermal stimuli, is theoretically justified and experimentally validated. Given that atomic vibrations fundamentally govern various response coefficients in a wide range of materials, and that thin films are typically deformed by substrates, it is anticipated that unprecedented responses can be generally attained through substrate‐induced control of atomic vibrations in thin films.

## Graphical abstract

Many material‐specific coefficients, which relate external stimuli and functional responses, are constants governed by atomic vibrations. This work demonstrates the concept of controlling such response coefficients by deformations, or strain, through strain‐induced changes in atomic vibrations. As a proof of concept, the dependence of the Curie constant on substrate‐imposed strain in SrTiO_3_ films is theoretically predicted and experimentally validated.

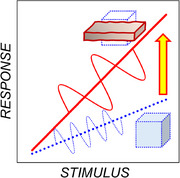

## 1 Introduction

Thin‐film materials represent a significant branch of modern materials research. Compared to bulk materials, whose chemical composition is traditionally adjusted to obtain desired properties, thin films provide distinct opportunities for innovative materials design. Here, we demonstrate a concept that leverages thin‐film phenomena to achieve unprecedented material responses, using ferroelectric films as an example.

Ferroelectrics, especially those of *AB*O_3_ perovskite type (here A and B stand for metal cations), exhibit strong responses to external electrical, mechanical, thermal, and optical stimuli as well as host coexisting robust effects.^[^ ^1^ ^]^ These properties ensure numerous applications of bulk ferroelectric ceramics and crystals.^[^ ^1^, ^2^, ^3^, ^4^, ^5^, ^6^, ^7^ ^]^ The application‐relevant stimulus‐response relationships are characterized by basic coefficients, which are constant for a given material and include, for instance, elastic constants, electrostriction coefficients, and the Curie constant. To obtain superior response coefficients, material compositions are varied.

Studies of thin ferroelectric films have been substantially progressing since the early 1990′s ^[^ ^8^, ^9^, ^10^ ^]^ to nowadays,^[^ ^11^, ^12^, ^13^, ^14^, ^15^, ^16^, ^17^, ^18^, ^19^, ^20^, ^21^ ^]^ leading to innovative devices and fundamental discoveries.^[^ ^22^, ^23^, ^24^ ^]^ Because a thin film is mechanically bound to the substrate or electrode layer on which it is grown, the film's material is deformed. Substrate‐imposed deformations, or strains, have been shown to affect crystal structure, polarization, domains, and phase transitions in ferroelectric films.^[^ ^26^, ^27^, ^28^, ^29^, ^30^, ^31^, ^32^ ^]^ Concurrently, intrinsic materials’ response coefficients are commonly considered as utterly unchangeable constants.^[^ ^1^, ^2^, ^3^, ^4^, ^5^, ^6^, ^7^, ^8^, ^9^, ^10^, ^11^, ^12^, ^13^, ^14^, ^15^, ^16^, ^17^, ^18^, ^19^, ^20^, ^21^, ^22^, ^23^, ^24^, ^25^, ^26^, ^27^, ^28^, ^29^, ^30^, ^31^, ^32^ ^]^

Here, we show that strains modify vibrations of atoms around their equilibrium positions in the crystal lattice, or lattice vibrations. Consequently, response coefficients, determined by lattice vibrations, can vary with strain, resulting in unique responses.

For our study, we have chosen SrTiO_3_ (STO) as the best explored archetypal representative of *AB*O_3_ perovskites belonging to ferroelectrics. We combined the state‐of‐the‐art first‐principles analysis and experimental investigations of STO films. We found that the frequencies and magnitudes of lattice vibrations depend on strain, leading to the strain‐dependent Curie constant – a strain effect not recognized before. The results indicate a way toward superior responses through strain‐controlled lattice vibrations for a wide range of materials.

## 2 Results and Discussion

### 2.1 Strain‐Dependent Lattice Vibrations and Curie Constant

Pure unstressed STO is paraelectric at all temperatures, possesses the ideal perovskite structure with the centrosymmetric cubic space group *Pm*‐3*m* (SG221) at *T* > 105 K, and acquires the tetragonal phase *I*4/*mcm* (SG140) at low *T* < 105 K. In strained STO films, the high‐temperature tetragonal phase is well established.^[^ ^26^, ^28^, ^33^ ^]^ In the paraelectric state, which is typical for STO and other *AB*O_3_ ferroelectrics at high temperatures, the real part of the dielectric permittivity *ε* obeys the Curie‐Weiss law [*ε* = *C_C_*/(*T* – *Θ*)], where *C_C_* and *Θ* are the Curie constant and temperature, respectively.^[^ ^34^ ^]^ Whereas the para‐to‐ferroelectric phase transition is manifested by the maximum permittivity at the transition temperature *T_m_*, the Curie–Weiss law is normally valid at temperatures by ≈50 K higher than *T_m_* ≈ *Θ*.^[^ ^34^ ^]^

**Figure 1:**
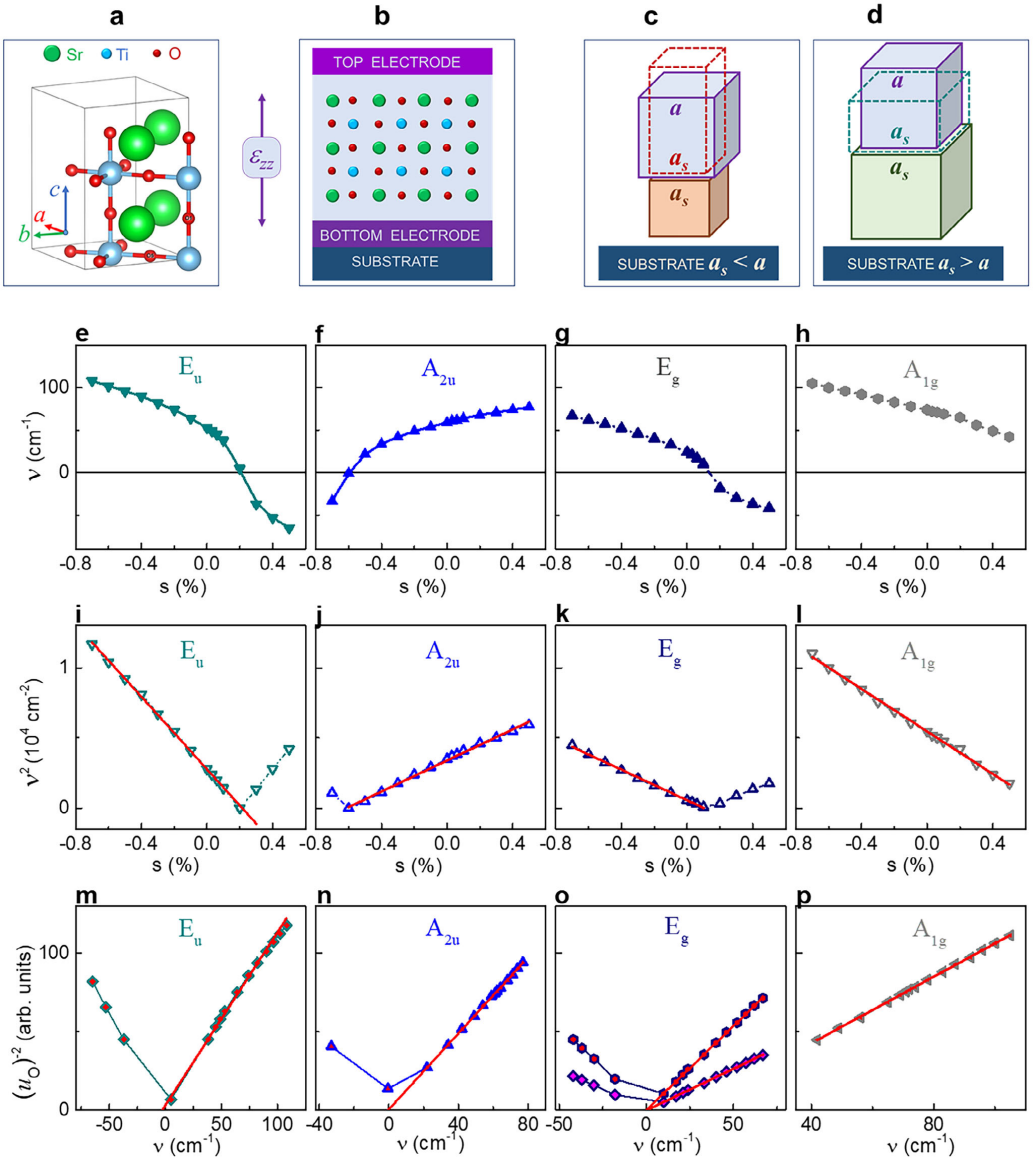
Schematics of the a) 20‐atom crystallographic unit cell of the low‐temperature tetragonal phase *I4/mcm* (SG 140) in STO and b) atomic planes in epitaxial (001) oriented STO film. c,d) Schematics of the unit‐cell deformations in the films (dashed lines) compared to the unstressed cell (blue cubes) for (c) compressive and (d) tensile substrates. e–h) Vibrational frequencies as a function of in‐plane strain for the lowest‐frequency modes E_u_, A_2u_, E_g_, and A_1g_ as marked on the plots. i–l) Squared vibrational frequencies as a function of in‐plane strain. Straight red lines show fits. m–p) Inverse square of oxygen displacements *u_O_* ^−2^ as a function of frequency. Displacements are along *x*‐ and *y*‐ axes in m) and p), *z*‐axis in n), *x*‐ and *y*‐ axes (lower line) and *z*‐axis (upper line) in o). Straight lines show fits.

Here, the theoretical analysis of strained STO films was performed within the linear combination of atomic orbitals approximation of density functional theory using the tetragonal SG140 structure as the computational model (**Figure** **1**a). The atomic SrO and TiO_2_ planes are parallel to the substrate surface in (001) oriented STO films (Figure 1b). The in‐plane lattice parameters of the film are clamped by the underlying substrate, leading to a biaxial in‐plane strain (Figure 1c,d). Therefore, in the calculations, the lattice parameters of the film, *a_s_* = *b_s_*, were varied compared to *a* = *b* in strain‐free STO and fixed for each value of the in‐plane strain *s* = (*a_s_*/*a* ‐1). The angles between the lattice axes were fixed to 90 degrees. The lattice parameter *c* and positions of all atoms were allowed to relax.

In the absence of strain (*s* = 0), the calculated lowest‐frequency vibrational modes are E_g_, E_u_, A_2u_, and A_1g_ with their frequencies *ν* being 24, 53, 59, and 74 cm^−1^, correspondingly (Table S1, Supporting Information). The vibrational frequencies strongly alter with strain (Figure 1e–h). The modes E_u_, A_2u_, and E_g_ become imaginary (*ν* < 0) beyond a range of strains (−0.6% < *s* < 0.2%), implying that with increasing in‐plane compressive strain to the magnitude of 0.6% or tensile strain to 0.2%, the film remains in the same paraelectric phase, whereas other, polar phases exist at larger strains. The modes E_u_ and A_2u_ are infrared‐active and produce the main contributions to the static dielectric permittivity (Figure S1, Supporting Information).

The squared frequencies of the modes are found to be linear functions of strain: [*ν* ^2^ ∝ *s*] (Figure 1i–l) that formally resembles the temperature‐dependent Cochran law [*ν* ^2^ ∝ *T*] in bulk STO.^[^ ^35^, ^36^ ^]^ However, here, the frequencies alter with strain, and the slopes of [*ν* ^2^ ∝ *s*] are positive for A‐modes but negative for E‐modes.

The strain‐dependent vibrational frequencies are crucial for the strain‐dependent Curie constant *C_C_*. The Curie constant *C_C_* is determined by the atomic displacements *u_k_* in the lattice vibrations: [*C_C_* ∝ {∑*_k_m_k_* (*u_k_*)^2^}^−1^], where *m_k_* is the mass of the atoms in the unit cell.^[^ ^35^, ^36^, ^37^, ^38^ ^]^ The sums ∑(*mu* ^2^) were calculated separately for Sr atoms, Ti atoms, O atoms, and for all atoms in total for the main four lowest‐frequency modes (Figure S2a–d, Supporting Information). The total sums are dominated by oxygen contributions, while those from Ti are an order of magnitude smaller and those from Sr – nearly two orders of magnitude smaller.

Thus, the lattice dynamics is mainly determined by vibrations of oxygen atoms, for which the atomic displacements *u_O_* and vibrational frequencies are found to relate as [*u_O_* ^−2^ ∝ *ν*] (Figure 1m–p). Because of the strain‐dependent frequencies and the frequency‐displacement correlations, the linear relationships [∑(*mu* ^2^))^−2^ ∝ *s*] persist within the paraelectric phase (Figure S2e–h, Supporting Information). This strain‐dependent behavior leads to the strain‐dependent Curie constant.

**Figure 2:**
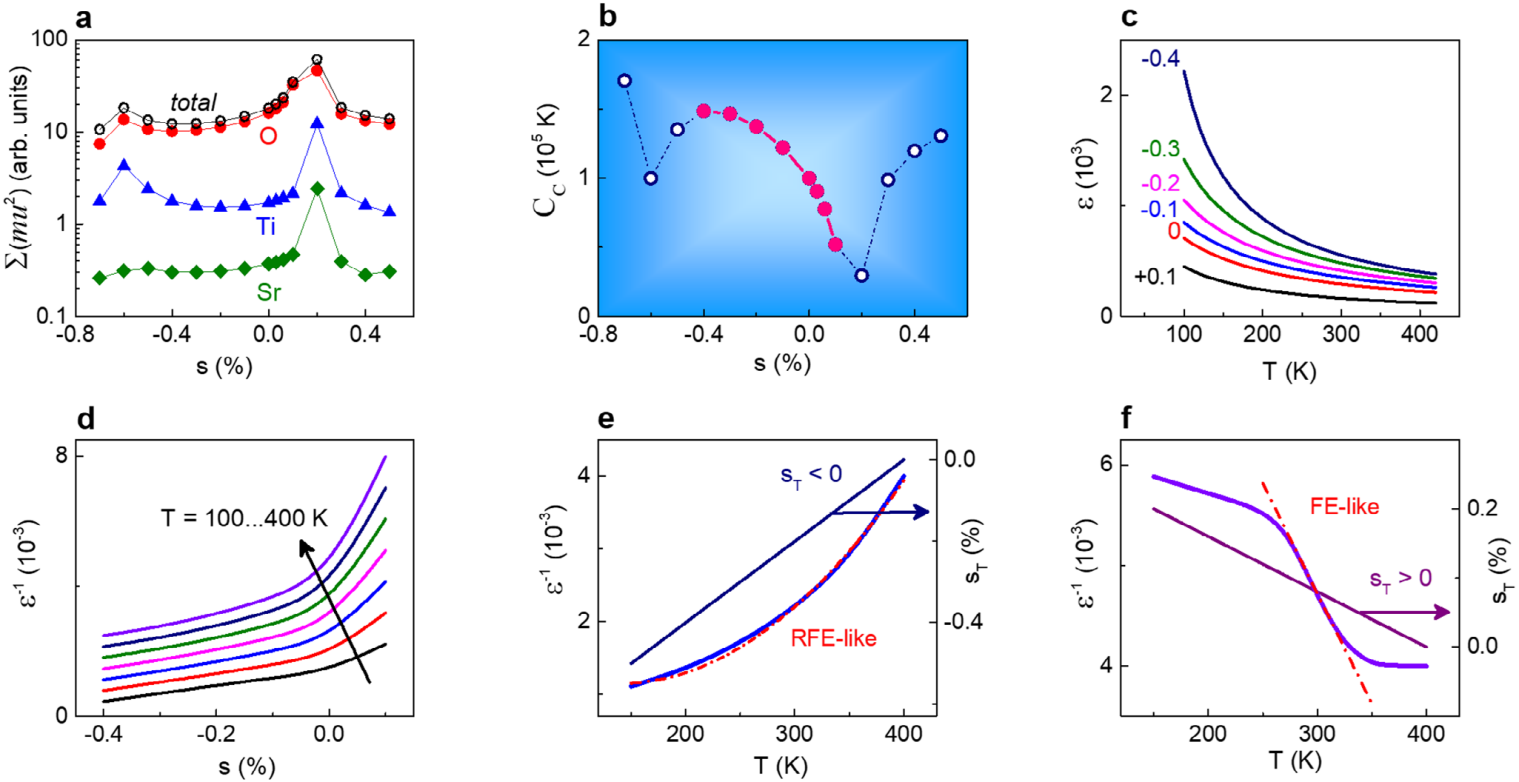
Calculated a) sums ∑(*mu* ^2^) for atomic vibrations and b) Curie constant *C_C_* as a function of strain. In (b), solid symbols correspond to the paraelectric phase. c) Estimated out‐of‐plane dielectric permittivity as a function of temperature for different in‐plane strains (in % as marked on the plots). d) Estimated inverse permittivity as a function of strain for different temperatures. The arrow shows the direction of temperature increase. e,f) Estimated inverse permittivity as a function of temperature in the presence of in‐plane (e) compressive and (f) tensile thermal strain *s_T_*, also shown on the plots. Dashed lines show fits to the (e) relaxor‐like [*ε* ^−1^ ∝ *T* ^2^] and (f) ferroelectric‐like [*ε* ^−1^ ∝ ‐*T*] behavior.

The Curie constant *C_C_* was obtained using the calculated strain‐dependent entire sum of all atomic vibrations in the lowest‐frequency modes (**Figure** **2**a) and assuming *C_C_* = 1.0 × 10^5^ K at zero strain *s* = 0 (Figure 2b). The strain‐dependent Curie constant varies between ≈1.5 × 10^5^ and ≈0.5 × 10^5^ K within the paraelectric phase: it grows from 1.0 × 10^5^ to ≈1.5 × 10^5^ K with increasing compressive strain from zero to 0.4% and falls to ≈0.5 × 10^5^ K for tensile strain of 0.1% only.

**Figure 3:**
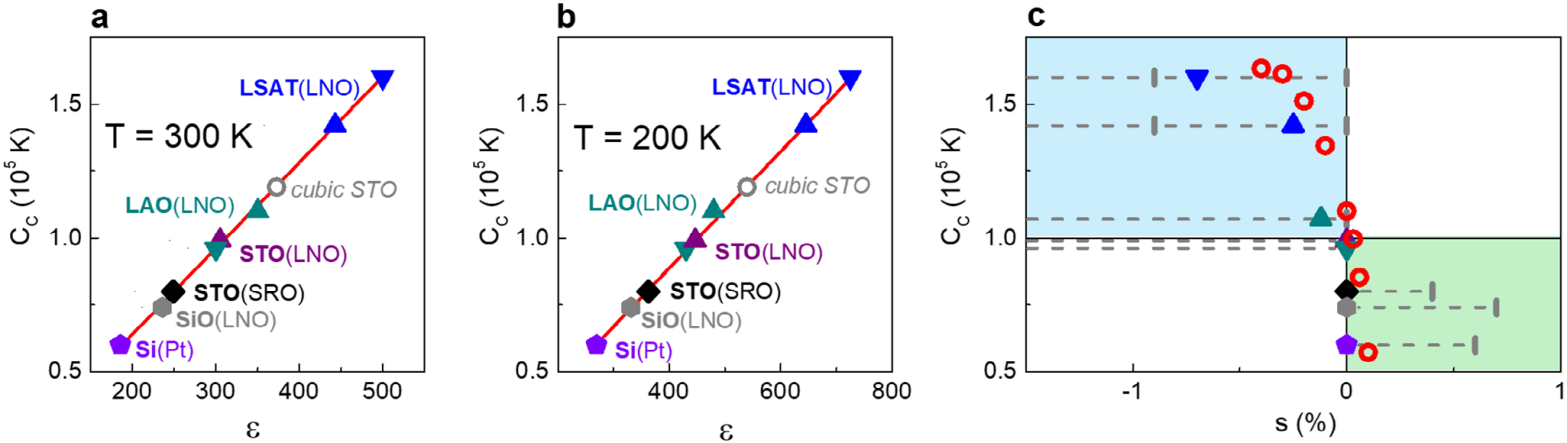
a,b) Relationships between the Curie constant and the dielectric permittivity at the temperature a) 300 K and b) 200 K inside STO films on different substrates (bottom electrodes). Data for the reference cubic STO crystal are shown for comparison. Straight lines show fits to [*C_C_* ∝ *ε*] with the slopes of (a) 310 K and (b) 220 K. c) The experimental (solid symbols) and theoretical (open symbols) intrinsic Curie constant as a function of strain in STO films. Dashed horizontal lines show the theoretical in‐plane strain for STO films on different substrates (bottom electrodes). Blue and green quadrants show regions on in‐plane compressive and tensile strains, correspondingly.

Notably, the Curie constant rapidly changes with strain at very small, near‐zero strains (**Figure** **3**b). The changes in *C_C_* by 20% can result from strain alterations of only ±0.03% around zero. Such strains in thin films are difficult to analyse using common laboratory equipment.

To briefly illustrate the role of the massive strain‐dependent variations in the Curie constant, the out‐of‐plane permittivity was calculated using [ε  = *C_C_*/(*T* – *Θ*)] with the found *C_C_* (Figure 2b), and considering *ε* = *ε_zz_* at *T* = 100 K (Figure S1, Supporting Information). As seen from Figure 2c, compressive strain enlarges permittivity, which rapidly grows on cooling due to the strain‐enhanced Curie constant. Conversely, tensile strain leads to a weakened temperature dependence and smaller magnitudes of permittivity due to the strain‐suppressed Curie constant. Also, because of the strain‐dependent intrinsic Curie constant, a non‐linear strain‐permittivity relationship can occur (Figure 2d). Furthermore, in the presence of thermal strain *s_T_* (arising due to film‐substrate mismatch in thermal expansion coefficients), the strain‐controlled Curie constant can modify the very character of the temperature dependence [*ε* ^−1^(*T*)]. For the in‐plane compressive thermal strain [*s_T_* < 0], our simulations show the relationship [*ε* ^−1^ ∝ *T* ^2^], that looks like that in relaxor ferroelectrics (RFE) (Figure 2e). For the in‐plane tensile thermal strain [*s_T_* > 0], the simulated curve [*ε* ^−1^ (*T*)] contains an increasing fraction that mimics the low‐temperature ferroelectric behavior (Figure 2f).

The theoretically unveiled effects of strain on the dielectric Curie–Weiss response are incredibly strong. Next, we present experimental verification of the theoretical predictions.

### 2.2 Curie–Weiss Behavior in STO Films

To experimentally validate the theoretical findings, we investigated the temperature‐dependent dielectric behavior and the Curie constant in diverse STO thin‐film capacitor stacks.

To ensure variations of strain in thin (≈150 nm) films of STO, the vertical capacitor stacks were grown using different substrates [(001)‐oriented SrTiO_3_ (STO), (LaAlO_3_)_0.3_(Sr_2_AlTaO_6_)_0.7_ (LSAT), and LaAlO_3_ (LAO), (0001)‐oriented SiO_2_, and Pt‐coated Si] and different bottom electrode layers [≈30‐nm‐thick SrRuO_3_ (SRO) or LaNiO_3_ (LNO), as well as commercially prepared ≈200‐nm‐thick Pt]. In such STO films, the theoretical room‐temperature in‐plane strain arising from mismatches in lattice parameters or in thermal expansion coefficients, is estimated between −2.9% and +0.8% (Note S2, Figure S3, Table S2, Figure S4, Supporting Information). The stacks were prepared by pulsed laser deposition, and the strain was additionally tailored through growth conditions.^[^ ^39^, ^40^, ^41^ ^]^ The films were single‐crystalline on STO, LSAT, and LAO, and polycrystalline on SiO_2_ and Si substrates (Note S2, Figures S5–S12 and Table S3, Supporting Information). The room‐temperature residual in‐plane strain in STO films (**Table** **1**) was evaluated considering the measured lattice parameters (Table S3, Supporting Information) and the theoretical estimations. A weak residual strain (marked by 0….) is likely although difficult to resolve experimentally. The negative in‐plane strain (highlighted in blue in Table 1) is compressive (as illustrated in Figure 1c) and the positive in‐plane strain (highlighted in green) is tensile (as illustrated in Figure 1d). The negative strain is expected to raise the Curie constant *C_C_*, whereas the positive – to diminish *C_C_* (as shown in **Figure** **3**c).

**Table 1:** Residual room‐temperature in‐plane strain in STO films in different stacks.

substrate	LSAT	LSAT	LAO	LAO	STO	STO	Si	SiO_2_
electrode	LNO	LNO	LNO	LNO	LNO	SRO	Pt	LNO
*s_a_*, %	−0.9^*^	−0.2	−0.1^*^	−0….	−0….	+0….	+0.1	+0.3

*For STO films prepared at an oxygen pressure of 10 Pa.^[^ ^40^ ^]^

The Curie–Weiss behavior [*ε* ^−1^ = *T*/*C_C_* – *Θ*/*C_C_*] was evidenced for the temperatures from ≈100 to ≈400 K in all stacks (Note S3, Figures S13–S18, Supporting Information). The experimentally determined intrinsic Curie constant ranges between 0.6 × 10^5^ and 1.6 × 10^5^ K (Table S4, Supporting Information).

The obtained spread in the Curie constant is massive and cannot be ascribed to an imaginable non‐STO fraction in the films. Straightforward estimations (Note S4 and Table S5, Supporting Information) show that the non‐STO fraction should comprise at least ≈40% of the film's volume to yield ± 50% alterations of the Curie constant. Such a tremendous volume fraction should have been manifested in substrate‐dependent films’ composition, which is not the case for our films, nor for pulsed laser deposition principally.

To further verify the intrinsic nature of the observed behavior, we inspected the Curie–Weiss relationship in the form [*ε* ∝ *C_C_*] across different films for different fixed temperatures (Note S5, Supporting Information). The experimental relationships between *ε* and *C_C_* are linear (Figure 3a,b). Furthermore, the slopes of [*C_C_* ∝ *ε*] practically coincide with the measurement temperatures as expected. These observations confirm the strain‐dependent intrinsic Curie constant.

The experimentally detected variations of *C_C_* are in excellent agreement with the theoretically predicted ones (Figure 3c). Compared to unstressed STO, the magnitude of *C_C_* strongly increases in the presence of compressive in‐plane strain (blue upper left quadrant in Figure 3c) but significantly diminishes in the presence of in‐plane tensile strain (green lower right quadrant in Figure 3c). The theoretically estimated and the experimentally determined variations of *C_C_* are huge, ± 50%.

Additionally, we examined the dielectric Curie–Weiss behavior in STO film subjected to the in‐plane thermal tensile strain, imposed by the sapphire substrate (Note S6, Supporting Information). The found deviation from the linear behavior [*ε* ^−1^ ∝ *T*] (Figure S21, Supporting Information) complies with the theoretical trend (Figure 2f).

Our systematic experiments confirm the theoretically anticipated variations of the Curie constant with strain. We note that experimental studies of the Curie constant are rare. Nevertheless, scattered Curie constants and frustration of the Curie–Weiss behavior have been detected before.^[^ ^42^, ^43^, ^44^, ^45^, ^46^, ^47^, ^48^ ^]^ Our experimental observations are consistent with earlier works from different groups.

Thus, our investigations show that substrate‐induced changes in lattice vibrations can lead to strain‐dependent films’ response constants. We demonstrate the strain‐dependent Curie constant, but also other response coefficients, which are regulated by lattice vibrations (e.g., coefficients of electrostriction), can vary with strain. Importantly, lattice vibrations determine diverse response constants in materials in general. Likewise, generally, thin films are mechanically deformed by substrates. Therefore, our findings indicate a universal possibility to tailor responses of thin films through substrate‐controlled lattice vibrations.

**Figure 4:**
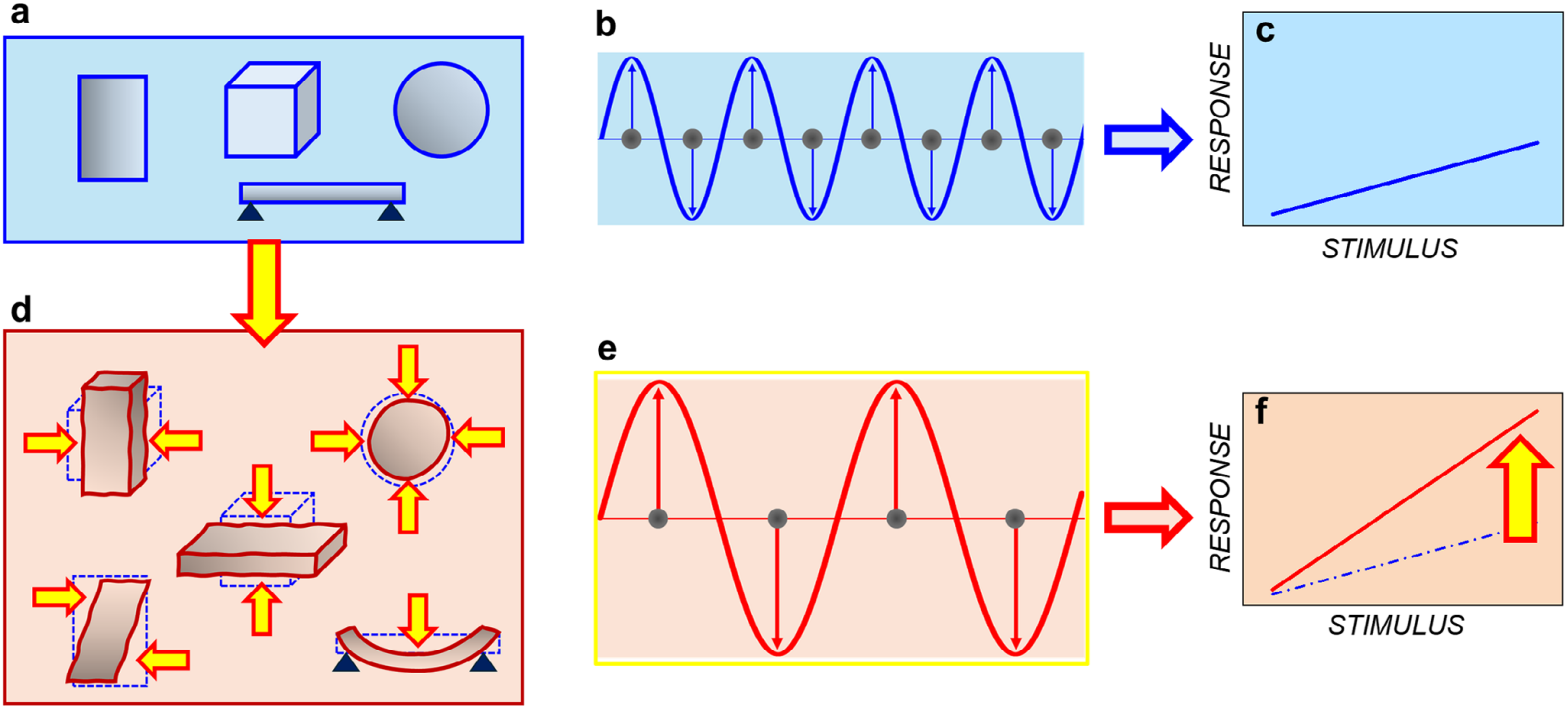
Schematics of strain‐controlled response coefficients: a) undeformed objects and b) atomic vibrations and c) response‐stimulus relationship therein; d) deformations, or strains in the objects and e) atomic vibrations and f) response‐stimulus relationship (in red) in the deformed objects. Strain (marked by the yellow arrow between (a) and (d)) induces changes in response coefficients (as marked by the yellow arrow in (f)) through changes in atomic vibrations (compare (e) with (b)).

On an even broader scale, our work suggests a concept of tailoring responses through deformation‐induced changes in atomic vibrations as schematically illustrated in **Figure** **4**. In undeformed objects (Figure 4a), atomic vibrations (Figure 4b) determine coefficients in various response‐stimulus relationships (exemplified as a slope of the response‐stimulus line in Figure 4c). Deformations, or strains (Figure 4d) alter atomic vibrations (compare Figure 4e with Figure 4b). Correspondingly, the response coefficients change (e.g., strain‐induced larger slope of the response line in Figure 4f). We anticipate that the concept can be applied to diverse objects (thin films, membranes, 2D materials, nanoparticles, nanotubes, biological cells, molecules, etc) and responses (electromechanical, electrical, optical, chemical, etc).

## 3 Conclusion

The substrate‐controlled strain‐dependent Curie constant and dielectric behavior are theoretically discovered through first‐principles calculations and experimentally validated in thin films of the representative archetypal perovskite oxide strontium titanate. The substrate‐induced strains are shown to significantly influence lattice vibrations, leading to response coefficients which are regulated by these vibrations, become strain‐dependent. The results justify a novel universal approach to achieving superior material responses through strain‐controlled lattice vibrations.

## 4 Experimental Section

### Computational Methods

The structural, vibrational, and dielectric properties of STO were calculated using the linear combination of atomic orbitals (LCAO) approximation of density functional theory (DFT), combined with the hybrid functional as implemented in the CRYSTAL17 computer code for quantum‐chemical simulations.^[^ ^49^, ^50^ ^]^ The B1WC global hybrid DFT‐HF exchange‐correlation functional, which combines the Wu‐Cohen WCGGA exchange functional with 16% (by default) of Hartree‐Fock (HF) exchange and the Perdew–Wang PWGGA correlation functional, was employed in this study. Gaussian‐type basis sets with Hay and Wadt's small core effective core pseudopotentials were used for Sr and Ti atoms, while an all‐electron basis set with exponents for d‐shells was applied for describing oxygen atoms.^[^ ^51^, ^52^, ^53^, ^54^ ^]^ The SG140 crystallographic structure was used as the computational model. The Wyckoff positions of the 20 atoms in the unit cell were Sr: 4b (0, 1/2, 1/4), Ti: 4c (0, 0, 0), OI: 4a (0, 0, 1/4), and OII: 8 h (1/2‐x, x, 0). The transverse optical (TO) vibrational frequencies and amplitudes, as well as the vibrational contributions to the static dielectric tensor, were calculated at the Γ‐point (the centre of the first Brillouin zone) within the harmonic approximation.

### Experimental Methods

Thin‐film capacitor stacks were prepared by pulsed laser deposition (PLD).^[^ ^39^, ^40^, ^41^ ^]^ Single‐crystal STO, LSAT, LAO, SiO_2_, and Al_2_O_3_, and Pt‐coated Si substrates were purchased from MTI Corp., USA. The Pt top electrodes (0.2‐2.0 mm^2^) were formed by PLD through a shadow mask. The proper stoichiometry of the films was confirmed by x‐ray fluorescence spectroscopy (XRF) on an Orbis PC Micro‐XRF spectrometer (EDAX, Ametek, data analysis by Orbis Vision), energy dispersive spectroscopy (EDS) on a scanning electron microscope FEI QUANTA 3D (Thermo Fisher Scientific, data analysis by GENESIS), and x‐ray photoelectron spectroscopy (XPS) on a NanoESCA instrument (Oxford Instruments Omicron Nanoscience, data analysis by CasaXPS). Crystal structure and lattice parameters in thin‐film stacks were inspected by high‐resolution x‐ray diffraction (HRXRD) using Cu Kα radiation on a SmartLab SE Multipurpose diffractometer (Rigaku Corp.) and a D8 DISCOVER diffractometer (Bruker Corp.)

The complex electrical impedance was measured on a NOVOCONTROL Alpha‐AN High‐Performance Frequency Analyzer and using Linkam cold/hot stages.

## Conflict of Interest

The authors declare no conflict of interest.

## Supporting information



Supporting Information

## Data Availability

The data that support the findings of this study are available in the supplementary material of this article.
