# Supplementary material for: Substrate‐Controlled Response Coefficients in Thin Films
Source: Adv Sci (Weinh). 2025 Aug 19;12(43):e05761. doi: 10.1002/advs.202505761 (PMC12631899; doi:10.1002/advs.202505761)
Supplement: Supplementary file 1 — Supporting Information [file ADVS-12-e05761-s001.pdf]

Supporting Information

Substrate-controlled response coefficients in thin films

M. Tyunina,[1,2*] L. L. Rusevich,[3] M. Savinov,[2] T. Kocourek,[2] O. Pacherova,[2] A. Dejneka,[2] E. A. Kotomin[3,4]

[1]*Microelectronics Research Unit, Faculty of Information Technology and Electrical Engineering, University of Oulu, P. O. Box 4500, FI-90014 Oulu, Finland*
[2]*Institute of Physics of the Czech Academy of Sciences, Na Slovance 2, 18221 Prague, Czech Republic*
[3]*Institute of Solid State Physics, University of Latvia, Kengaraga Str. 8, LV-1063 Riga, Latvia*
[4]*Max Planck Institute for Solid State Research, Heisenberg Str. 1, Stuttgart D-70569, Germany*

SI Note 1

Lattice vibrations and dielectric function

The transverse optical (TO) vibrational frequencies and vibrational contribution to the dielectric tensor were calculated within the harmonic approximation in the center of the first Brillouin zone (at the Γ-point) using optimized (equilibrium) geometry. The complex dielectric function $\varepsilon(v)$ is a sum of the electronic (high-frequency) and ionic (vibrational) components: $\varepsilon(v) = \varepsilon_{el} + \varepsilon_{vib}(v)$, where v is the frequency of the TO vibrational modes. The dielectric function was calculated within the CRYSTAL code using a classical dispersion relation of the Drude-Lorentz model [1–3]:

$$\varepsilon(\nu) = \varepsilon_{el} + \sum_j \frac{f_j \nu_j^2}{\nu_j^2 - \nu^2 - i\nu\gamma_j} , \quad \text{(S1)}$$

where ε_{el} is the high-frequency dielectric constant, v_j, f_j and γ_j are the frequency of the jth TO IR-active vibrational mode, the oscillator strength and the damping factor, respectively [1]. From Eq. (1), the static dielectric tensor is equal to

$$\varepsilon(0) = \varepsilon_{el} + \sum_j f_j = \varepsilon_{el} + F , \quad \text{(S2)}$$

where the vibrational contribution F is the sum of the oscillator strengths. Wherein, the oscillator strength f_j of the jth IR-TO normal mode for an isotropic crystal is calculated by means of the expression

$$f_j = \frac{4\pi}{V} \frac{Z_j^2}{\nu_j^2} , \quad \text{(S3)}$$

where V is the cell volume and Z_j^2 is the mass-weighted effective mode Born charge [3,4]. The electronic contribution ε_{el} is calculated in CRYSTAL through the coupled perturbed Hartree-Fock/Kohn-Sham (CPHF/CPKS) scheme, adapted for periodic systems [5,6].

Table S1. Vibrational modes in unstressed tetragonal STO.

Mode symmetry	Frequency ν, cm^{-1}	IR activity	Raman activity	intensity, arb. units
E_g	24.2	-	+	
E_u	53.0	+	-	1
A_{2u}	59.3	+	-	0.5
A_{1g}	73.7	-	+	
E_g	145.4	-	+	
B_{2g}	147.2	-	+	
E_u	173.4	+	-	
A_{2u}	174.7	+	-	
E_u	259.1	+	-	
B_{1u}	262.3	-	-	
A_{1u}	452.7	-	-	
B_{2g}	453.2	-	+	
E_g	453.5	-	+	
E_u	453.6	+	-	
A_{2g}	495.5	-	-	
B_{1g}	497.5	-	+	
E_u	554.2	+	-	0.2
A_{2u}	555.6	+	-	0.1
A_{2g}	872.8	-	-	

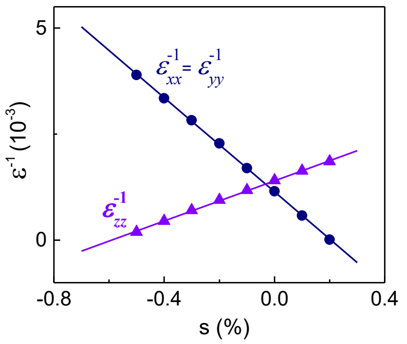

Figure S1. Calculated inverse permittivity components.

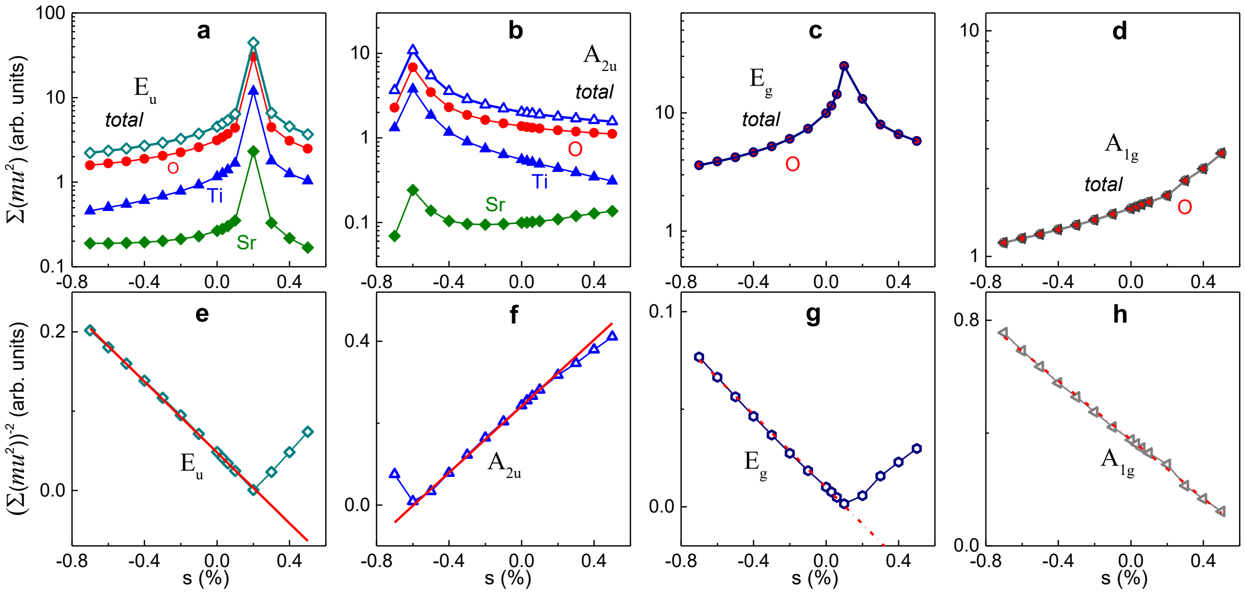

Figure S2. Calculated (**a-d**) sums $\sum(mu^2)$ of atomic vibrations and (**e-h**) inverse squared of the total sums $(\sum(mu^2))^{-2}$ as a function of strain in the lowest frequency E_u, A_{2u}, E_g, and A_{1g} modes. Straight lines show fits in (e-h).

SI Note 2

Thin-film capacitor stacks

2.1. Lattice misfit strain

To ensure variation of strain in STO films, thin-film capacitor stacks were grown on different substrates and using different bottom electrode layers. The theoretical in-plane strain s_a, originating from the film-substrate (or film-electrode) misfit in lattice parameters, was estimated as $s_a = (a_{SUB}/a_0 - 1)$. Here a_{SUB} and a_0 are the lattice parameters of unstressed (pseudo)cubic perovskite (sub)cell of the underlying substrate or bottom electrode and of the top film (STO or bottom electrode), correspondingly. The estimated room-temperature strains in thin films of LNO, STO, and SRO are shown as a function of lattice parameter of underlying layers in Figure S2.

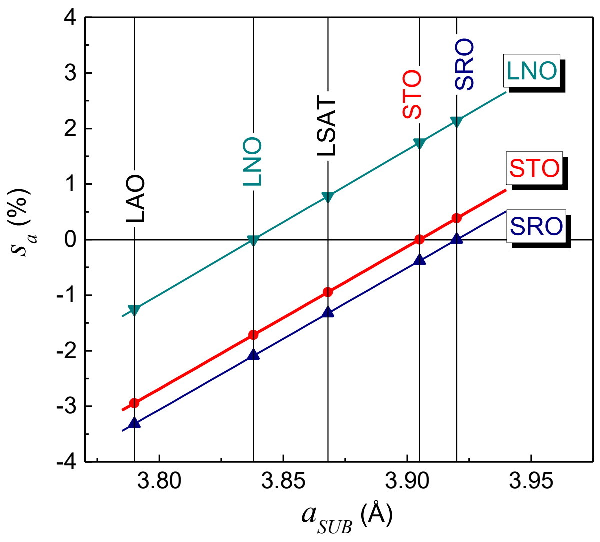

Figure S3. Theoretical room-temperature in-plane lattice misfit strain s_a (in %) for pseudocubic perovskite cells of LNO, STO, and SRO films coherent to (pseudo)cubic perovskite cells of LAO, LNO, LSAT, STO, and SRO substrates. The in-plane lattice parameters of substrates are marked by vertical lines.

Because elastic energy of a strained film increases with increasing film's thickness, there is a critical thickness for the relaxation of misfit strain. In epitaxial films of ABO_3 perovskite oxides,

strain relaxation occurs through different mechanisms including classical development of morphological instabilities and formation of dislocations, as well as perovskite-specific tilting/rotation of BO_6 octahedra and formation of domains. Therefore, the critical thickness for films in Figure S2 is difficult to estimate theoretically, although qualitatively, the critical thickness is known to decrease with increasing strain magnitude and to depend on film's material. Typically, relaxation of strains of 0.5 % begins at thicknesses of ~10 nm. In thick enough films, the film-substrate misfit strain can fully relax, i.e. residual strain becomes zero. Considering the theoretical misfits and the complete misfit relaxation as limiting cases, the in-plane strains in epitaxial STO films can be in the ranges listed in Table S2.

Table S2. Theoretical room-temperature in-plane strain s_a (in %) for STO films in different stacks.

**electrode.substrate**	**s_a, %**
**SRO.STO**	0…+0.4
**LNO.STO**	-1.9…0
**LNO.LSAT**	-1.9…0
**LNO.LAO**	-2.9…0

2.2. Thermal strain

For thin films grown at high temperatures, a mismatch between thermal expansion coefficients of the substrate and film's materials leads to a temperature-dependent thermal strain. The in-plane thermal strain s_{at} is equal to $s_{at} = (\alpha_0 - \alpha_{SUB})\cdot(T_D - T)$, where α_0 and α_{SUB} are the thermal expansion coefficients of the film and substrate materials, correspondingly, and T_D is the film deposition temperature. The thermal expansion coefficients of STO, LSAT, and LAO are approximately the same resulting in a negligible thermal strain in STO films in stacks on STO, LSAT, and LAO substrates. The thermal expansion mismatch is significant for STO films on Si and SiO_2. The thermal in-plane and out-of-plane strains in STO on Si and SiO_2 were estimated considering the thermal expansion coefficients of Si [7] (Figure S3a) and SiO_2 (5.5×10^{-7} K^{-1}) and T_D = 973 K (Figures S3b and 3c). The out-of-plane strain s_{ct} was calculated as $s_{ct} = -(2c_{12}/c_{11})\cdot s_{at}$, where $c_{12} = 1.03\times10^{11}$ N/m^2 and $c_{11} = 3.48\times10^{11}$ N/m^2 are the elastic constants of STO. The thermal strain is alleviated in the presence of appropriate bottom electrode and/or buffer layers.

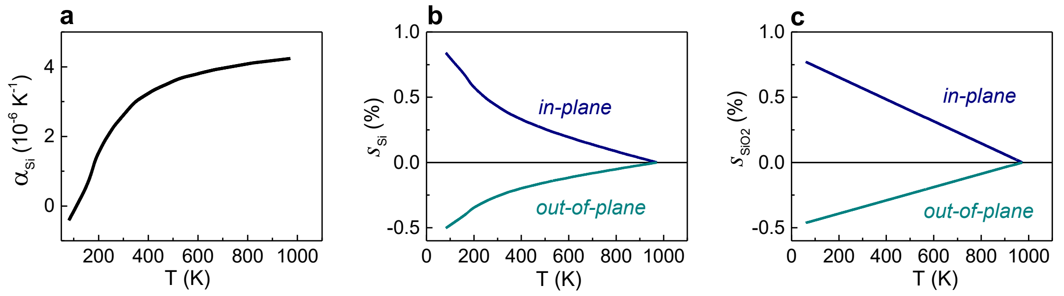

Figure S4. (a) Thermal expansion coefficient of Si [7] and (b, c) theoretical in-plane and out-of-plane thermal strain as a function of temperature in STO films on (b) Si substrate and on (c) SiO_2 substrate.

2.3. XRD scans

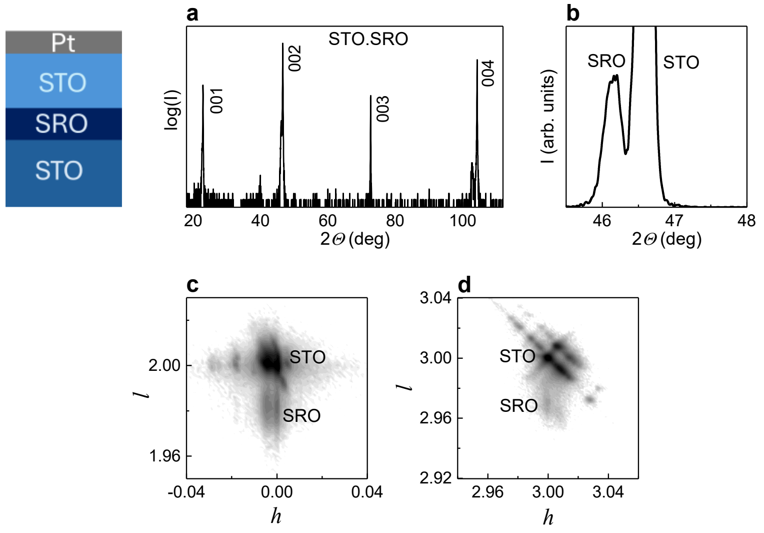

Figure S5. XRD (a, b) ω-2Θ scans and (c, d) reciprocal space maps for stack of STO on SRO/STO (shown schematically on the left). In (c, d), the coordinates are shown in reciprocal lattice units of STO.

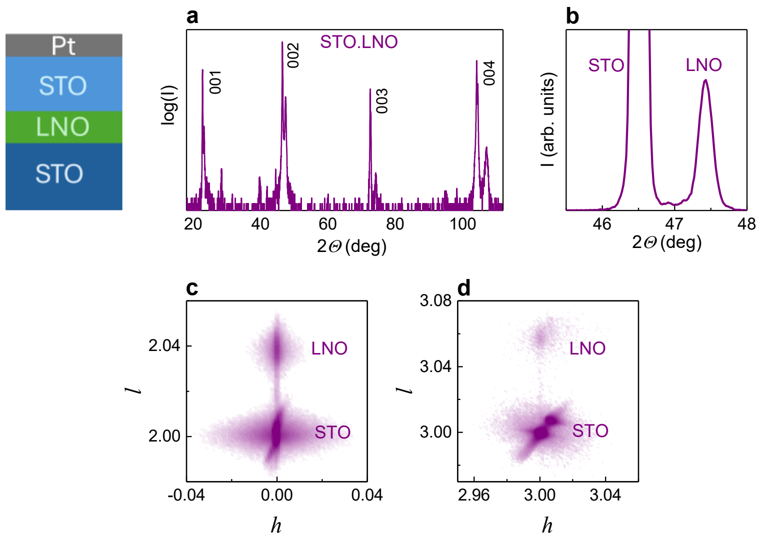

Figure S6. XRD (a, b) ω-2Θ scans and (c, d) reciprocal space maps for stack of STO on LNO/STO (shown schematically on the left). In (c, d), the coordinates are shown in reciprocal lattice units of STO.

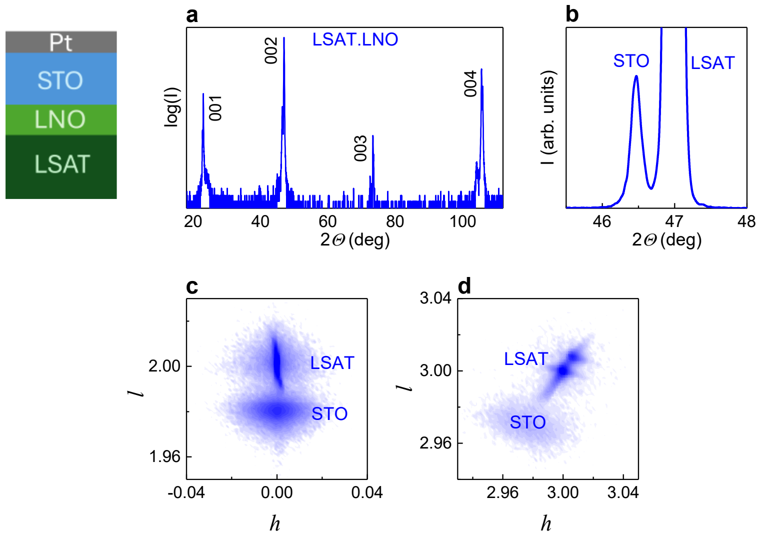

Figure S7. XRD (a, b) ω-2Θ scans and (c, d) reciprocal space maps for stack of STO on LNO/LSAT (shown schematically on the left). In (c, d), the coordinates are shown in reciprocal lattice units of LSAT.

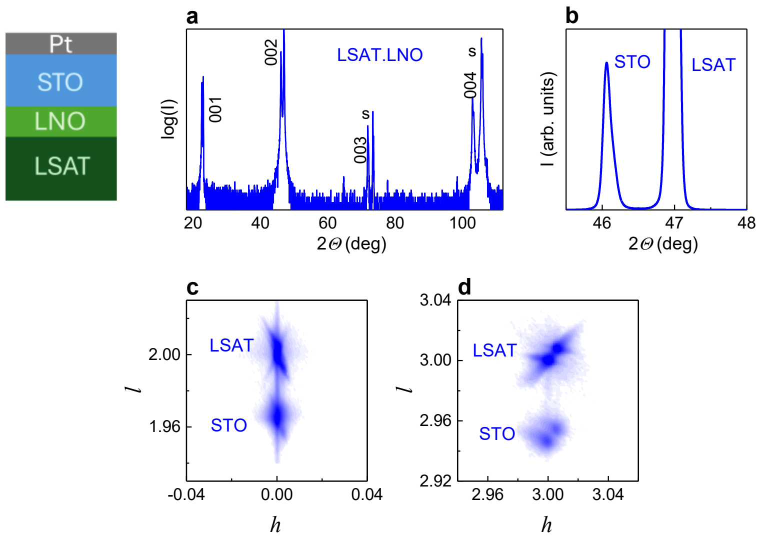

Figure S8. XRD (a, b) ω-2Θ scans and (c, d) reciprocal space maps for stack of STO (10 Pa) on LNO/LSAT (shown schematically on the left). In (c, d), the coordinates are shown in reciprocal lattice units of LSAT.

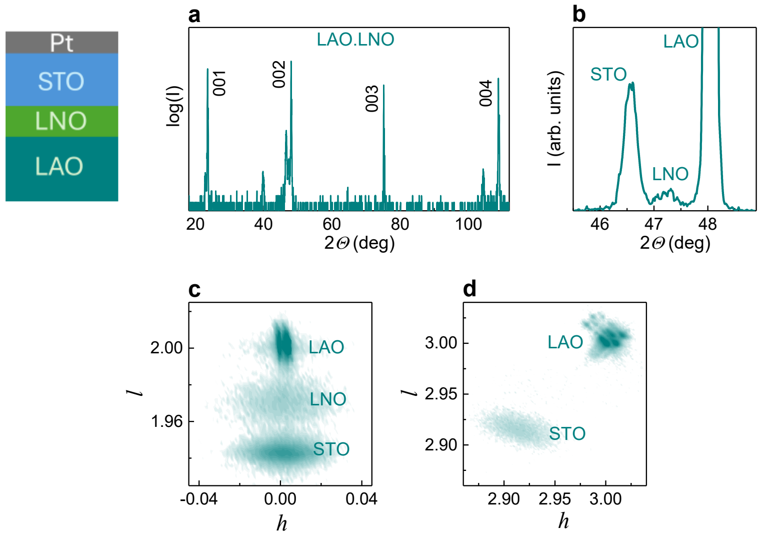

Figure S9. XRD (a, b) ω-2Θ scans and (c, d) reciprocal space maps for stack of STO on LNO/LAO (shown schematically on the left). In (c, d), the coordinates are shown in reciprocal lattice units of LAO.

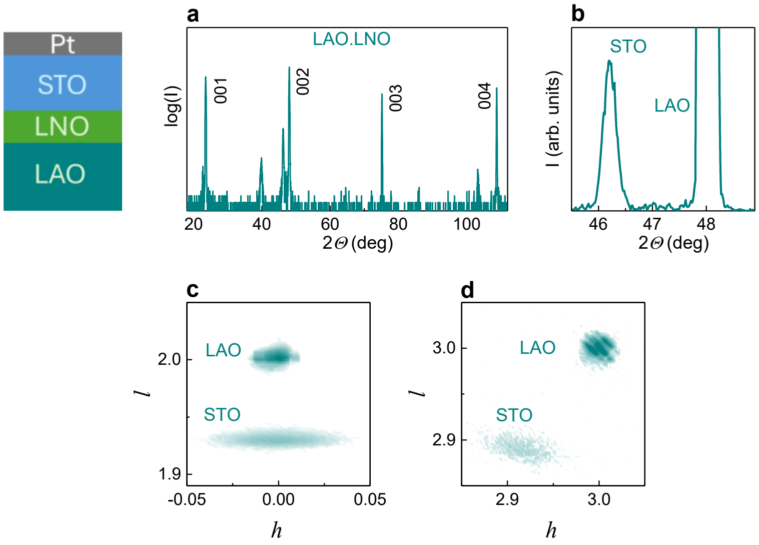

Figure S10. XRD (a, b) ω-2Θ scans and (c, d) reciprocal space maps for stack of STO (10 P) on LNO/LAO (shown schematically on the left). In (c, d), the coordinates are shown in reciprocal lattice units of LAO.

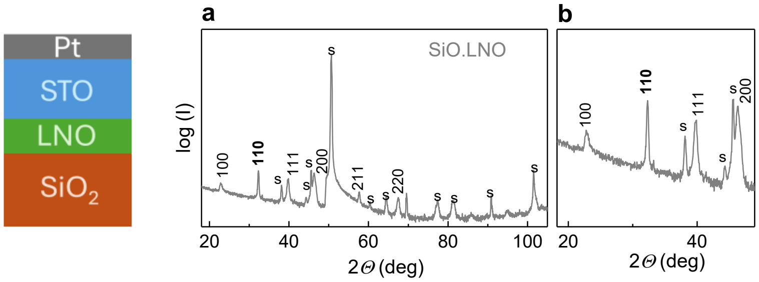

Figure S11. XRD (a, b) ω-2Θ scans for stack of STO on LNO/SiO_2 (shown schematically on the left). The peaks from substrate are marked by “s”.

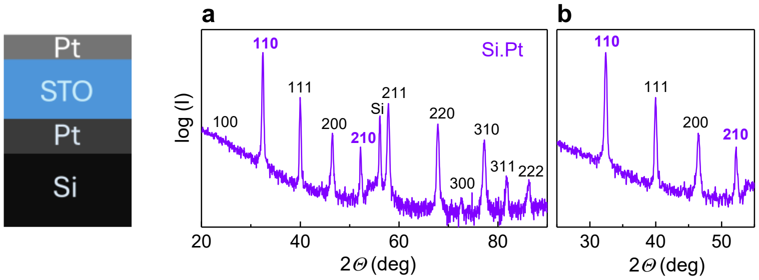

Figure S12. XRD (a, b) ω-2Θ scans for stack of STO on Pt/Si (shown schematically on the left). The peaks from substrate are marked by “s”.

Table S3. Measured average room-temperature lattice parameters of electrodes and (001)-oriented STO films (fractions) in different stacks. Diffractions from the STO films and STO substrates overlap (lattice parameters are shown in brackets). Stars mark stacks with the STO films grown at 10 Pa oxygen.

**substrate**	**electrode**		**(001)STO**	
**(electrode)**	in-plane	out-of-plane	in-plane	out-of-plane
**STO(SRO)**	3.905	3.945	(3.905)	(3.905)
**STO(LNO)**	3.905	3.830	(3.905)	(3.905)
**LSAT(LNO)**	3.868	3.830	3.896	3.907
**LAO(LNO)**	3.792	3.845	3.905	3.905
**LSAT(LNO)**	3.868	3.830	3.868*	3.938*
**LAO(LNO)**	3.792	3.845	3.900*	3.932*
**Si/SiO_2/Ti(Pt)**				3.902
**SiO_2(LNO)**				3.898

SI Note 3

Dielectric behavior

3.1. Impedance analysis

The real part of the dielectric permittivity ε was extracted from the complex impedance measured in the STO capacitor stacks with the Pt top electrodes (Fig. S10 and expressions S4-S8).

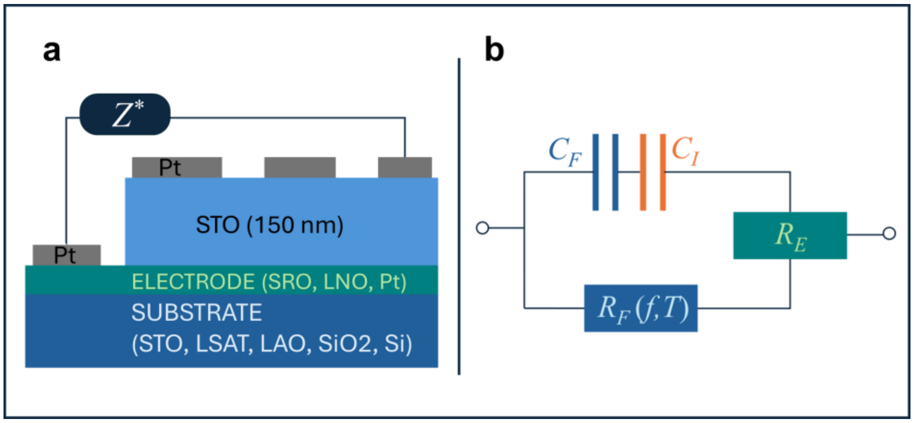

Figure S13. Schematics of (a) cross-section and (b) equivalent circuit of STO thin-film capacitor stack.

Equivalent-circuit model

Z^* - complex impedance of the capacitor
C_F – capacitance of STO film
C_I – temperature-independent STO-electrode interfacial capacitance
R_F – resistance describing dielectric losses and charge transport in STO
R_E – resistance describing bottom electrode, probes, wires
$\omega = 2\pi f$, where f is the frequency
d – thickness of STO film
d_I – thickness of interfacial capacitance layer: $d_I << d$
ε – measured permittivity in capacitor stack
ε_F – permittivity inside STO film
ε_I – temperature-independent permittivity in interfacial capacitance layer
C_C – Curie constant in STO film
Θ_F – Curie temperature inside STO film

Θ – apparent measured Curie temperature in capacitor stack

$$Z^* = R - iX = \left[R_E + \frac{R_F}{1+(\omega C R_F)^2}\right] - i\left[\frac{\omega C R_F^2}{1+(\omega C R_F)^2}\right] \quad \text{(S4)}$$

$$\frac{1}{C} = \frac{1}{C_F} + \frac{1}{C_I} \quad \text{(S5)}$$

$$\frac{1}{\varepsilon} \approx \frac{1}{\varepsilon_F} + \frac{d_I}{\varepsilon_I d} = \frac{1}{\varepsilon_F} + \Delta_I \quad \text{(S6)}$$

$$\frac{T-\Theta}{C_C} = \frac{T-\Theta_F}{C_C} + \Delta_I \quad \text{(S7)}$$

$$\Theta = \Theta_F - C_C\Delta_I \quad \text{(S8)}$$

3.2. Conductivity of STO films

The electrical AC conductivity of STO films was very small [8] so that the film's resistance R_F was large and did not disturb analysis of the capacitance and the dielectric permittivity in the studied frequency-temperature range. The conductivity was smaller than 10^{-8} S/cm at frequency 1 kHz. Furthermore, the films deposited at lower oxygen pressure exhibited even smaller conductivity (Figure S13).

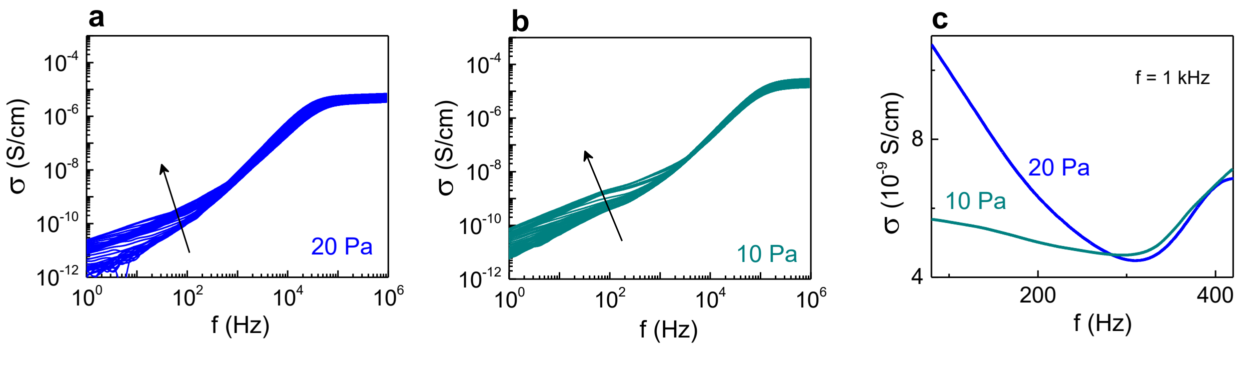

Figure S14. Small-signal AC conductivity σ as a function of (a, b) frequency at different temperatures and (c) temperature at frequency 1 kHz in STO films deposited at different oxygen pressures on LNO/LSAT. Arrows show directions of temperature increase from 80 to 500 K in (a, b). Oxygen pressure is marked on the plots.

3.3. Curie-Weiss behavior

Because of relatively large resistances of oxide thin-film electrodes (R_E in Fig. S13), there is a cut-off frequency in the dielectric response of the capacitor stacks (Figs. S15a-f and Fig. S16a-b), that limits the range of frequencies for permittivity analysis. The temperature dependence of the measured permittivity was inspected for frequency of 1 kHz (Figs. S15g-l and Figs. S16c-d).

The inverse permittivity is found to linearly increase with temperature evidencing the validity of the Curie-Weiss behavior $\varepsilon = C_C/(T - \Theta)$ or $\varepsilon^{-1} = T/C_C - \Theta/C_C$ for the temperatures from ~100 to ~400 K in the stacks (Figs. S15m-r and Figs. S16e-f). Due to the film-electrode interfacial capacitance (Fig. S13), the measured permittivity ε and the apparent Curie temperature Θ in the stacks are lower than the intrinsic ones inside the films, whereas the Curie constant in the stack is equal to the film's intrinsic one (expressions S5-S8) [9, 10].

The experimentally determined intrinsic Curie constant ranges between 0.6×10^5 K and 1.6×10^5 K in the STO films. As a reference, we obtained $C_C = 1.2\times10^5$ K and $\Theta = -25$ K in the cubic unstressed STO crystal (Fig. S17a). We note that the fitted negative Θ for bulk STO crystals or ceramics is often adjusted to positive values using the modified Curie-Weiss law $\varepsilon = \varepsilon_L + C_C/(T - \Theta)$ [11]. In our study, the Curie constant for the modified fit is found to be by ~0.3×10^5 K smaller than that for the regular fits both in the crystal (Fig. S17b) and in the films (Fig. S18). For further analysis, we consider the simpler regular fits. The experimentally determined intrinsic Curie constants are listed in Table S4.

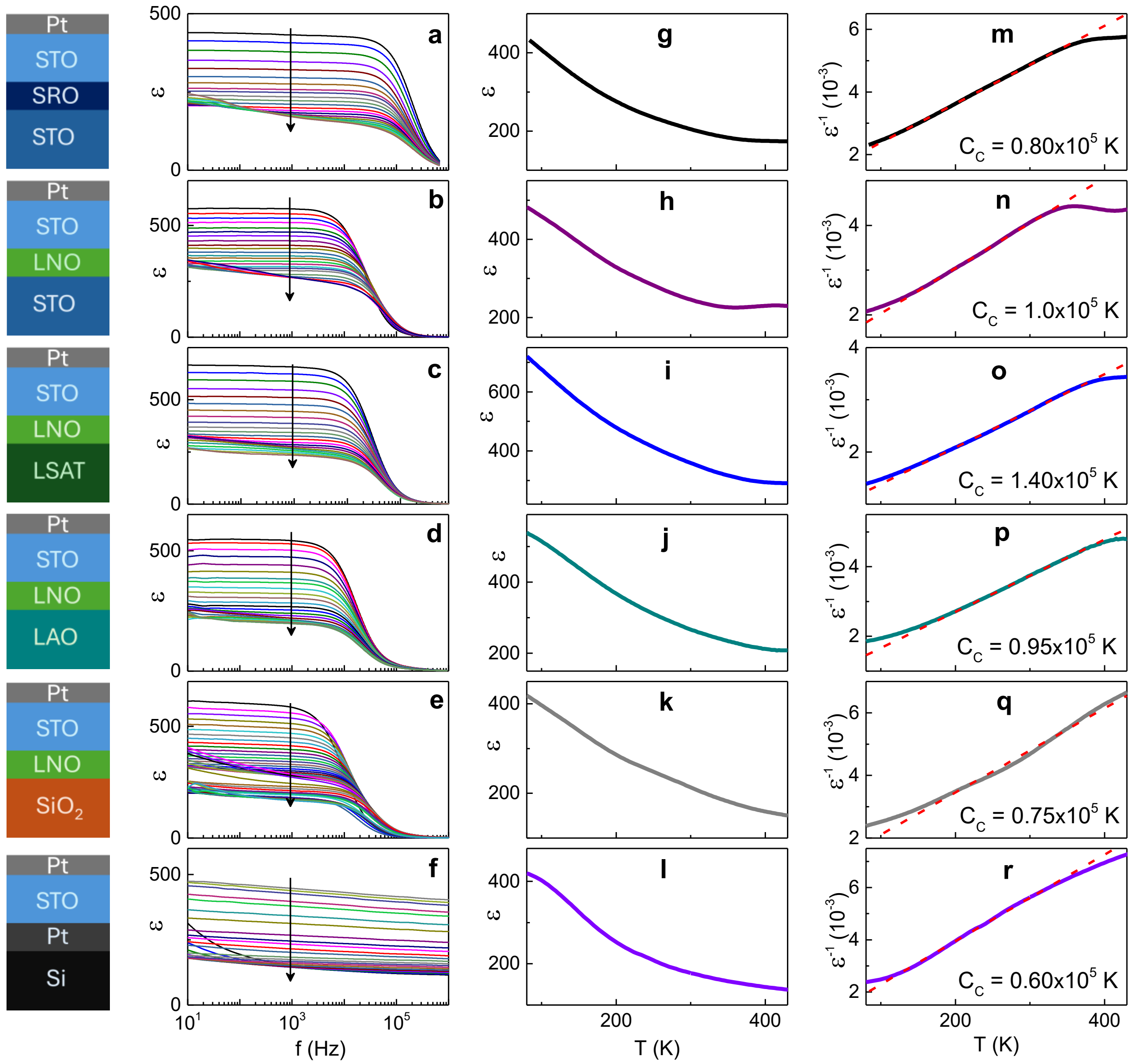

Figure S15. (a-l) The real part of the dielectric permittivity ε as a function of (a-f) frequency at various temperatures and (g-l) temperature at frequency of 1 kHz in different STO thin-film capacitor stacks (shown schematically on the left). Arrows in (a-f) show directions of temperature increase from 80 to 500 K. (m-r) Inverse permittivity ε^{-1} as a function of temperature for frequency of 1 kHz in different STO thin-film capacitor stacks. Dashed lines in (m-r) show fits to the Curie-Weiss law. The corresponding Curie constants C_C are also shown.

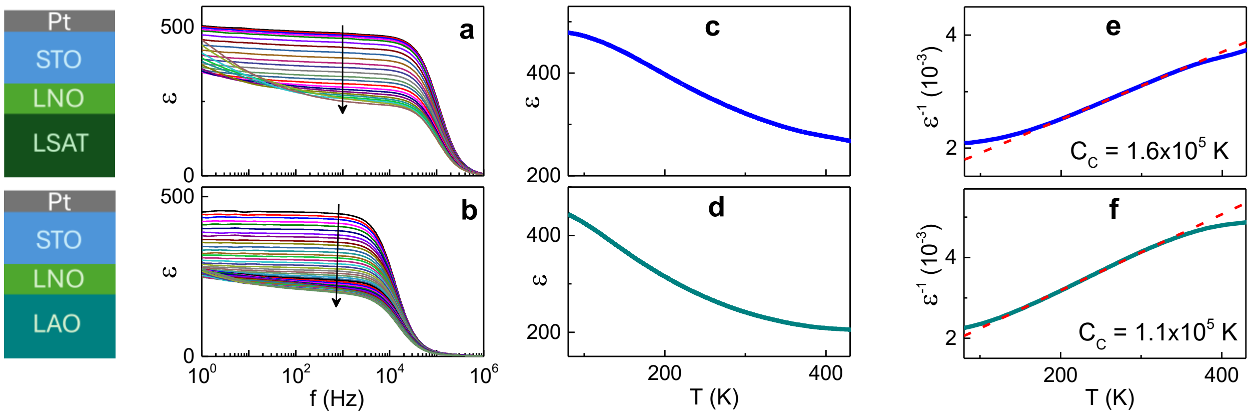

Figure S16. (a-d) The real part of the dielectric permittivity ε as a function of (a-b) frequency at various temperatures and (c-d) temperature at frequency of 1 kHz in different STO thin-film capacitor stacks (shown schematically on the left). STO films were deposited at 10 Pa oxygen. Arrows in (a-b) show directions of temperature increase from 80 to 500 K. (e-f) Inverse permittivity ε^{-1} as a function of temperature for frequency of 1 kHz in different STO thin-film capacitor stacks. Dashed lines in (e-f) show fit to the Curie-Weiss law. The extracted Curie constants are also shown.

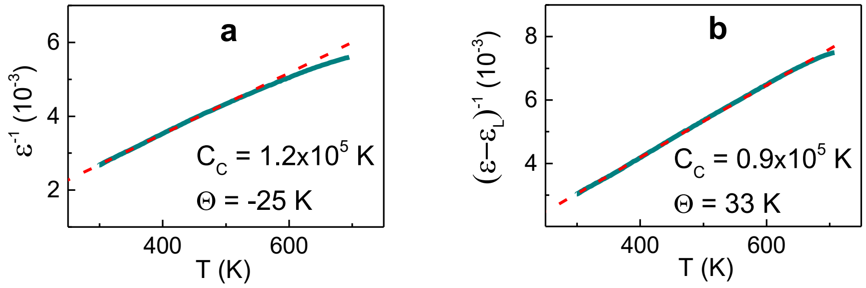

Figure S17. (a) Inverse permittivity ε^{-1} and (b) modified inverse permittivity $(\varepsilon - \varepsilon_L)^{-1}$ as a function of temperature for frequency of 500 kHz in the reference STO crystal. Dashed lines show fits. The extracted Curie constants and Curie temperatures are also shown.

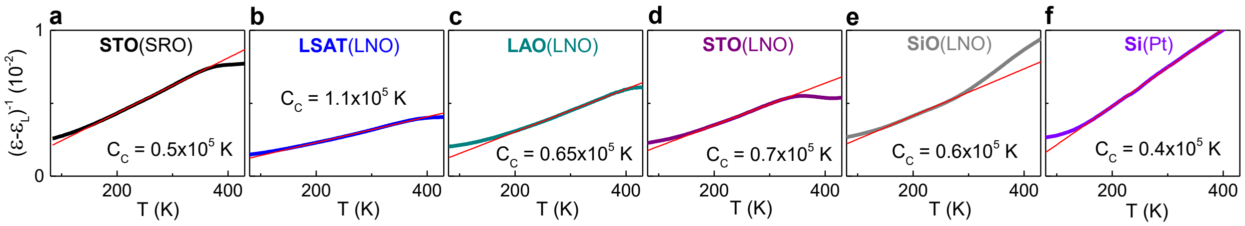

Figure S18. Modified inverse permittivity $(\varepsilon - \varepsilon_L)^{-1}$ as a function of temperature in different STO stacks as marked on the plots. Dashed lines show fits. The extracted Curie constants are also shown.

Table S4. The Curie constants C_C extracted from the fits to the regular Curie-Weiss laws in different STO thin-film stacks and reference crystal.

**SUBSTRATE** (ELECTRODE)	C_C, 10^5 K
**LSAT**(LNO)	**1.40**
**LSAT**(LNO)*	**1.60**
**STO**(LNO)	**1.00**
**LAO**(LNO)	**0.95**
**LAO**(LNO)*	**1.10**
**STO**(SRO)	**0.80**
**SiO_2**(LNO)	**0.75**
**Si**(Pt)	**0.60**
crystal	**1.20**

SI Note 4

Theoretical effects of non-STO inclusions on the apparent Curie constant

For inclusions of non-STO layer(s) inside the film

C , d , C_C – capacitance, thickness, and the apparent Curie constant
C_{STO}, d_{STO} , C_{CSTO} – capacitance, thickness, and the Curie constant of STO layer
C_{NON} and d_{NON} - capacitance and thickness of non-STO layer
Δ_I and X_I – temperature-independent terms

$$\frac{1}{C} = \frac{1}{C_{STO}} + \frac{1}{C_{NON}} + \frac{1}{C_I} \tag{S9}$$

$$d = d_{STO} + d_{NON} \tag{S10}$$

$$\frac{d}{\varepsilon} = \frac{d_{STO}}{\varepsilon_{STO}} + \frac{d_{NON}}{\varepsilon_{NON}} + \Delta_I \tag{S11}$$

$$\frac{dT}{C_C} = \frac{d_{STO}T}{C_{CSTO}} + \mathrm{X}_I \tag{S12}$$

$$\frac{C_C}{C_{CSTO}} = 1 + \frac{d_{NON}}{d_{STO}} \tag{S13}$$

For inclusions of non-STO columns inside the film

C , S , C_C – capacitance, capacitor area, and apparent Curie constant
C_{STO}, S_{STO} , C_{CSTO} – capacitance, total area, and Curie constant of STO columns
C_{NON} and S_{NON} - capacitance and total area of non-STO columns
Δ_I and X_I – temperature-independent terms

$$\frac{1}{C} = \frac{1}{C_F} + \frac{1}{C_I} \tag{S14}$$

$$C_F = C_{STO} + C_{NON} \tag{S15}$$

$$S = S_{STO} + S_{NON} \tag{S16}$$

$$\frac{T}{SC_C} \approx \frac{T}{S_{STO}C_{CSTO}} + \mathrm{X}_I \tag{S17}$$

$$\frac{C_C}{C_{CSTO}} = 1 - \frac{S_{NON}}{S_{STO}} \tag{S18}$$

For inclusions of non-STO boundaries around spherical STO grains

C_C – apparent Curie constant
C_{CSTO} - Curie constant of STO inside the grains
D – thickness of the grain boundary

R – radius of STO material inside the grain (R = 100 nm as an example here)
The ratio is calculated according to [12]:

$$\frac{C_C}{C_{CSTO}} \approx 1 - \frac{D}{R+D} . \qquad \text{(S19)}$$

Table S5. The ratio of the apparent total Curie constant, C_C, to the intrinsic one in STO, C_{CSTO}, in the presence of non-STO fraction in the form of horizontal layers, vertical columns, or grain boundaries of spherical grains.

**non-STO fraction, %**	**C_C/C_{CSTO} layers**	**C_C/C_{CSTO} columns**	**C_C/C_{CSTO} boundaries**
**0**	1.00	1.00	1.00
**10**	1.11	0.88	0.96
**20**	1.25	0.75	0.92
**30**	1.42	0.57	0.88
**40**	1.66	0.33	0.84

SI Note 5

Different capacitor stacks

Good linear fits to $\varepsilon \propto C_C$ are obtained for the permittivity directly measured in the capacitor stacks (Fig. S19).

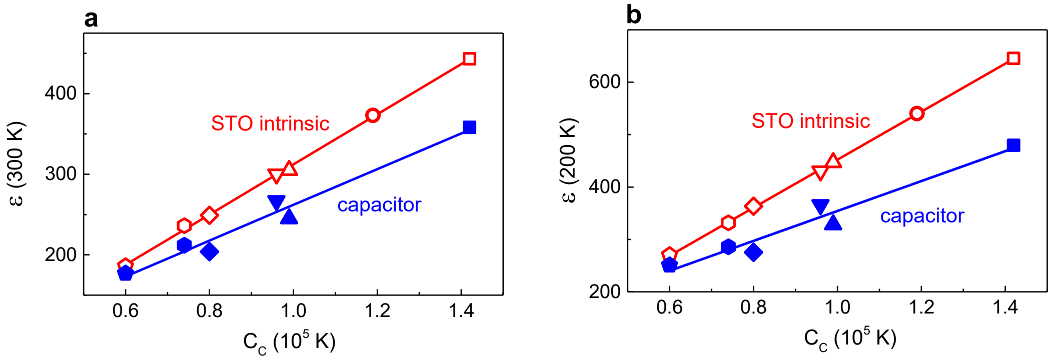

Figure S19. Relationships between the Curie constant and the dielectric permittivity at the temperature (a) 300 K and (b) 200 K in different capacitor stacks (marked as capacitor) and inside STO films (marked as STO intrinsic). Straight lines show fit to ($\varepsilon \propto C_C$).

The intrinsic permittivity inside the films can be estimated considering the Curie temperature in the films being equal to that in the crystal (expressions S6-S8).

For the slopes of the linear fits to $C_C \propto \varepsilon$:
d – thickness of STO film
d_I – thickness of interfacial capacitance layer: $d_I \ll d$
ε – measured permittivity in capacitor stack
ε_F – permittivity inside STO film
ε_I – temperature-independent permittivity in interfacial capacitance layer
C_C – Curie constant in STO film
Θ_F – Curie temperature in STO film
Θ – apparent measured Curie temperature in capacitor stack

The relationship inside the film:

$$C_C = \varepsilon_F(T - \theta_F) \qquad \text{(S20)}$$

The apparent relationship in the capacitor stack:

$$C_C = \varepsilon(T - \theta) = \frac{\varepsilon_F \varepsilon_I^I}{\varepsilon_F + \varepsilon_I^I}(T - \theta) \tag{S21}$$

$$\varepsilon_I^I \approx \frac{\varepsilon_I}{d_I} d \tag{S22}$$

SI Note 6

Effect of thermal strain

The role of thermal strain in the dielectric behavior was inspected in the ~150-nm-thick STO film grown on (0001)Al_2O_3 (sapphire) substrate using $SrRuO_3$ (SRO) bottom electrode. The linear thermal expansion coefficient of sapphire dramatically drops from ~9×10^{-6} K^{-1} at T = 1000 K to nearly zero at T = 50 K (Fig. S20a) [13]. Therefore, the STO film on sapphire is subjected to the substrate-induced in-plane tension. The theoretical in-plane thermal tensile strain grows from 0 % at the film's deposition temperature to ~ 0.9 % on cooling to 50 K (Fig. S20b).

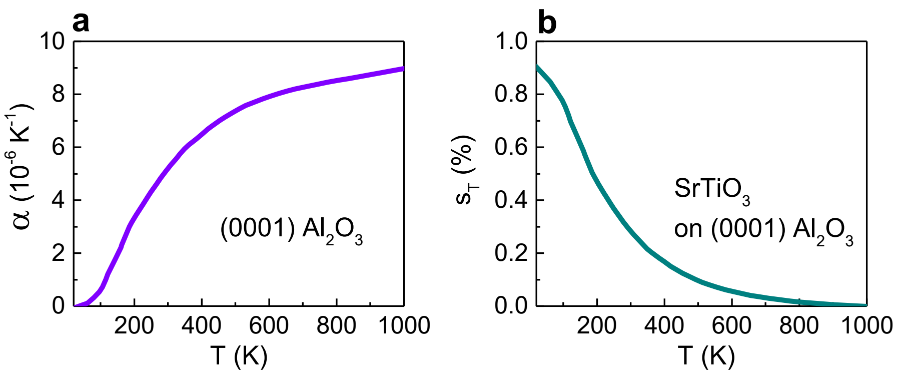

Figure S20. (a) Thermal expansion coefficient of sapphire and (b) the theoretical thermal strain in STO on sapphire as a function of temperature.

In the STO/SRO/sapphire stack, the measured inverse permittivity deviates from the linear Curie-Weiss behavior [$\varepsilon^{-1} \propto T$] at approximately 250 K (Figs. S21d-e). The temperature range for the deviation is very broad, at least 170 K. Therefore, the deviation cannot be related to a strain-induced ferroelectric transition. Concurrently, the deviation temperature of ~250 K is significantly higher than that of ~50 K, at which quantum fluctuations start causing a deviation from the Curie-Weiss behavior in STO crystals [14]. The observed deviation (Figs. S21d-e) is consistent with the strain-dependent Curie constant. It is worth noting that the high-temperature Curie constant extracted from the linear fraction of [$\varepsilon^{-1} \propto T$] (Fig. S21d) is very small and in excellent agreement with that determined by terahertz spectroscopy of the in-plane dielectric response [15].

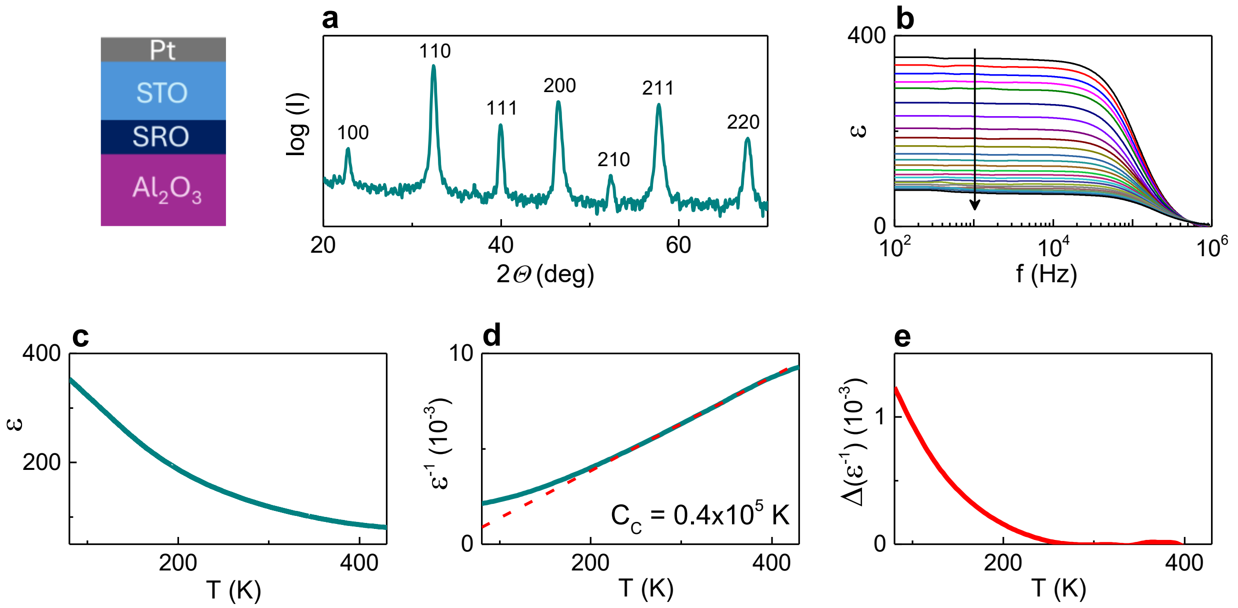

Figure S21. Stack of STO on SRO/Al_2O_3 (shown schematically on the left). (a) XRD ω-2Θ scan. (b-c) The real part of the dielectric permittivity ε as a function of (b) frequency at various temperatures and (c) temperature at frequency of 1 kHz. Arrow in (b) shows direction of temperature increase from 80 to 500 K. (d) Inverse permittivity ε^{-1} as a function of temperature for frequency of 1 kHz. Dashed line shows fit to the Curie-Weiss law. (e) The difference between the measured and fitted inverse permittivity as a function of temperature.

References

[1] Dovesi, R., Saunders, V. R., Roetti, C., Orlando, R., Zicovich-Wilson, C. M., Pascale, F., et al., *CRYSTAL17 User's Manual*, University of Torino, Torino, 2017.

[2] Decius, J. C., Hexter, R. M. *Molecular Vibrations in Crystals*, McGraw-Hill, New York, 1977.

[3] Dovesi, R., et al, The IR vibrational properties of six members of the garnet family: A quantum mechanical ab initio study. *Am. Miner.* **96**, 1787–1798 (2011).

[4] Demichelis, R. et al. The infrared spectrum of ortho-enstatite from reflectance experiments and first-principles simulations. *Mon. Not. R. Astron. Soc.* **420**, 147–154 (2012).

[5] Hurst, G. J. B., Dupuis, M., Clementi, E. Ab initio analytic polarizability, first and second hyperpolarizabilities of large conjugated organic molecules: applications to polyenes C_4H_6 to $C_{22}H_{24}$. *J. Chem. Phys.* **89**, 385–395 (1988).

[6] Kirtman, B., Gu, F. L., Bishop, D. M. Extension of the Genkin and Mednis treatment for dynamic polarizabilities and hyperpolarizabilities of infinite periodic systems. I. Coupled perturbed Hartree-Fock theory. *J. Chem. Phys.* **113**, 1294–1309 (2000).

[7] Okada, Y., Tokumaru, Y. Precise determination of lattice parameter and thermal expansion coefficient of silicon between 300 and 1500 K. *J. Appl. Phys.* **56**, 314 (1984).

[8] Tyunina, M., Savinov, M., Pacherova, O., Kocourek, T., Yudin, P., Dejneka, A. Strain-induced orders-of-magnitude reduction of hopping conductivity in epitaxial SrTiO3 films. *APL Mater.* **13**, 031101 (2025).

[9] Zhou, C., Newns, D. M. Intrinsic dead layer effect and the performance of ferroelectric thin film capacitors. *J. Appl. Phys.* **82**, 3081 (1997).

[10] Pertsev, N. A., Dittmann, R., Plonka, R., Waser, R. Thickness dependence of intrinsic dielectric response and apparent interfacial capacitance in ferroelectric thin films. *J. Appl. Phys*. **101**, 074102 (2007).

[11] Rupprecht, G. and Bell, R. O. Dielectric Constant in Paraelectric Perovskites. *Phys. Rev.* **135**, A748 (1964).

[12] Emelyanov, A.Yu., Pertsev, N.A., Hoffmann-Eifert, S., Bottger, U., Waser, R. Grain-Boundary Effect on the Curie-Weiss Law of Ferroelectric Ceramics and Polycrystalline Thin Films: Calculation by the Method of Effective Medium. *J. Electrocer.* **9**, 5 (2002).

[13] White, G.K., Minges, M.L. Thermophysical properties of some key solids: An update. *Int. J. Thermophys.* **18**, 1269 (1997).

[14] Barrett, J. H. Dielectric constant in perovskite type crystals. *Phys. Rev.* **86**, 118 (1952).

[15] Zhukova, E. S. et al. Terahertz ferroelectric soft mode in weakly doped $SrTiO_3$: M thin films (M=Mn, Ni, Fe, Co), *J. Alloys Compd.* **976**, 173255 (2024).
